# Optimisation of the HS-SPME/GC-MS Approach by Design of Experiments Combined with Chemometrics for the Classification of Cretan Virgin Olive Oils

**DOI:** 10.3390/metabo12020114

**Published:** 2022-01-25

**Authors:** Artemis Lioupi, Ioannis Sampsonidis, Christina Virgiliou, Vassiliki T. Papoti, Kyriaki G. Zinoviadou, Apostolos Spyros, Georgios Theodoridis

**Affiliations:** 1 Laboratory of Analytical Chemistry, School of Chemistry, Aristotle University of Thessaloniki, GR-54124 Thessaloniki, Greece; liouarte@chem.auth.gr (A.L.); cr_virgi@hotmail.com (C.V.); 2 Biomic AUTh, Center for Interdisciplinary Research and Innovation (CIRI-AUTH), Balkan Center B1.4, 10th km Thessaloniki-Thermi Rd, P.O. Box 8318, GR-57001 Thessaloniki, Greece; isampsonides@yahoo.gr; 3 FoodOmicsGR Research Infrastructure, AUTh Node, Center for Interdisciplinary Research and Innovation (CIRI-AUTH), Balkan Center B1.4, 10th km Thessaloniki-Thermi Rd, P.O. Box 8318, GR-57001 Thessaloniki, Greece; 4 Department of Nutritional Sciences and Dietetics, International Hellenic University, GR-57400 Thessaloniki, Greece; 5 Department of Food Science and Technology, Perrotis College, American Farm School, GR-55102 Thessaloniki, Greece; vpapot@afs.edu.gr (V.T.P.); kzinov@afs.edu.gr (K.G.Z.); 6 NMR Laboratory, Department of Chemistry, University of Crete, Voutes Campus, P.O. Box 2208, GR-71003 Heraklion, Crete, Greece; aspyros@chemistry.uoc.gr

**Keywords:** gas chromatography-mass spectrometry (GC-MS), headspace-solid phase microextraction (HS-SPME), olive oil, volatile organic compounds (VOCs), volatilomics, foodomics, metabolomics

## Abstract

A headspace-solid phase microextraction/gas chromatography-mass spectrometry (HS-SPME/GC-MS) method was developed herein for the analysis of virgin olive oil volatile metabolome. Optimisation of SPME conditions was performed by Design of Experiments (DoE) and Response Surface Methodology (RSM) approaches and factors, such as sample volume, sample stirring, extraction temperature and time, and desorption temperature and time, were examined to reach optimal microextraction conditions. The potential of the optimised method was then investigated for its use in the classification of Cretan virgin olive oil samples with the aid of multivariate statistical analysis. Certain markers were identified with significance in the geographical classification of Cretan extra-virgin olive oil (EVOO) samples. In total, 92 volatile organic compounds were tentatively identified and semi-quantified, and the data obtained confirm that the method is robust, reliable, and analytically powerful for olive oil classification.

## 1. Introduction

Extra virgin olive oil is one of the most significant ingredients of the Mediterranean diet, and its consumption has been significantly increased due to its high and special nutritional and sensory characteristics [1]. Virgin olive oil (VOO) is classified to quality categories as described by official standards (e.g., Codex Alimentarius, International Olive Council, and European Union) where sensory assessment is also included [2]. The sensory quality of VOO is determined by a panel test that evaluates positive and negative descriptors, according to European Union (EU) criteria (EU regulations) [3,4]. Olive oils possessing the fruity attribute and lacking any sensory defects can be categorized as “extra virgin”, whilst the existence and severity of sensory defects are used to verify whether the oil could be categorized as “virgin” or lampante [5]. Volatile compounds in olive oil can be linked to both positive and negative sensory characteristics and have a major effect on the oil’s quality and, as a result, on consumer preference. Hence, determining the VOO volatile fraction is important to ensure consumer protection, but also to avoid unfair competition, which can destabilize the market and disrupt regional/national economies.

In Greece, over 30 olive varieties are mainly produced [6]. Although Greece ranks third in the world in VOO production, the high-quality potential of Greek olive oils has been scarcely examined, and such information has not been communicated to the market. Crete is one of the main olive oil-producing areas in Greece and two oil cultivars, Koroneiki and Tsounati, mainly originate from there. Koroneiki, one of the most well-known and appraised olive cultivars in the world, is the predominant olive oil cultivar in Greece, accounting for about 60% of the country’s olive oil production (approximately 250,000 t) [6]. The Tsounati variety (also referred to as “Athinolia” or “Mastoeidis”) is less exploited and is cultivated in specific areas of Peloponnese, mainly in south Lakonia, Argolida, as well as in western Crete [7]. The quality potential of both examined cultivars can be partially revealed from the quality awards they gain from international competitions (e.g., Athena International Olive Oil Competition). It is necessary to verify the quality standards of Greek traditional high-added-value VOO to protect the final consumer from non-authentic food products and to help Greek food producers, suppliers, and service establishments compete in national and international markets [8,9,10].

Gas chromatography-mass spectrometry (GC-MS) has been commonly applied for the analysis of volatile organic compounds (VOCs) in EVOO, with an emphasis given in biomarker-detection, authenticity control, and issues associated with food quality and safety [11,12,13]. Headspace solid-phase microextraction (HS-SPME) has a number of advantages over other extraction methods, including automation and simplicity. Due to these advantages, it is usually the method of choice, with many applications in the field [14,15,16]. However, for this type of analysis, it is important to identify the role of all the possible factors that can influence the extraction of volatile compounds. In the present publication, sample volume, sample stirring, extraction temperature and time, and desorption time are the factors examined to reach optimal microextraction conditions. According to our knowledge, there are only two studies that focus on the optimisation of HS-SPME parameters by utilising advanced statistical techniques such as Design of Experiments (DOE) for VOO volatile compound analysis [17,18]. The presented approach is the first that optimises five HS-SPME factors simultaneously for EVOO analysis.

The overall goal was the development of an HS-SPME/GC-MS method for the analysis of a VOO volatile profile. For this purpose, an optimisation of SPME conditions was performed using DOE and Response Surface Methodology (RSM) approaches. The method was then applied for the analysis of VOOs originating from different regions of Crete, Southern Greece. This study was focused on the potential use of the optimised HS-SPME/GC-MS method combined with chemometrics for sample classification. The method proved to be robust, enabling the identification and semi-quantification of VOO VOCs.

## 2. Results and Discussion

### 2.1. Optimisation of SPME Extraction Conditions

**Figure 1:**
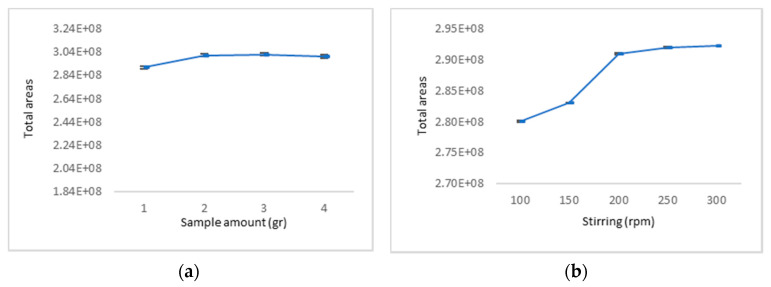

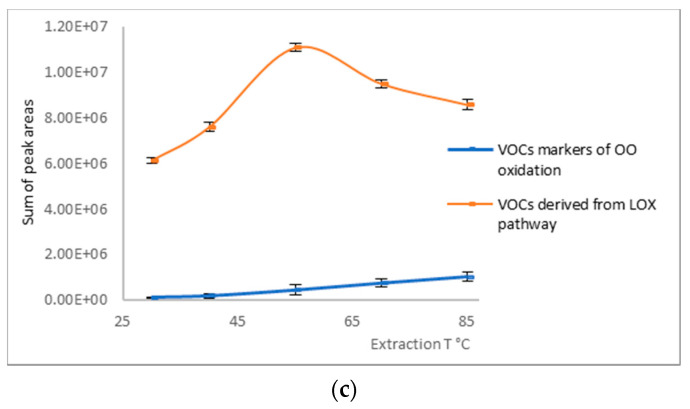
Influence of (**a**) sample amount, (**b**) stirring speed on total areas, and (**c**) extraction temperature on selected VOCs. (E + 08 = × 10^8^).

SPME sorption is dependent among other parameters on the sample volume, and it has been observed that extraction efficiency is improved when the volume of HS in the vial is minimised [19]. However, the extraction efficiency of volatile compounds may show a non-linear relation toward the sample volume due to fiber overload. Hence, the sample mass of the sample was examined under four different quantities (1.000, 1.850, 3.000, and 4.000 g). As illustrated in Figure 1a, sample amounts of 1.850 and 3.000 g improved extraction efficiency. However, a further increase from 3.000 to 4.000 g of sample had a negative effect on the extraction efficiency, yielding an optimal sample amount of 1.850 g.

For the optimisation of stirring, four different stirring speeds (100, 150, 200, 250, and 300 rpm) were evaluated. As shown in Figure 1b, after sample stirring at 250 rpm, no further improvement in extraction efficiency was observed. Hence, a stirring speed of 250 rpm was used for further experiments.

Although included in the DOE-RSM optimisation as a studied factor, a preliminary screening experiment was performed to determine the behaviour of the most critical HS-SPME condition for EVOO analysis, which is extraction temperature. Selection of an appropriate extraction temperature is crucial to minimise the oxidation of the olive oil sample inside the SPME vial, preventing the release of unwanted oxidation components [20]. More specifically, in unheated or heated-at-a-lower-temperature (50 °C) olive oil samples, a higher content of alcohols, esters, and terpenes has been observed compared to oils heated at higher temperatures; in the latter, higher amounts of aldehydes and carboxylic acids were found [21]. During oxidation off-flavor compounds produced due to fatty acid deterioration can be detected [22]. Consequently, the initially pleasant sensory characteristics of the oil attributed to compounds derived from the lipoxygenase (LOX) pathway eventually give way to unpleasant sensory attributes due to volatile compound deterioration. The screening of this parameter was performed by monitoring the total peak area of the selected components that are indicative of olive oil oxidation, while also monitoring components that are generally desirable according to the literature [21,22,23,24]. The selected compounds that indicate increased oil oxidation and must be kept at a minimum during sample heating were heptanal, 2-heptenal, octanal, and 2,4-heptadienal. The compounds that were selected as desirable for the analysis and contribute to its green odour notes [25] were 3-hexenal, (2*E*)-hexenal, 2-hexene-1-ol, hexan-1-ol, and 1-penten-3-one.

The study of extraction temperature showed an increase for both the desirable and undesirable oxidation components (Figure 1c). As indicated, there is a steep increase for the desirable components maximizing at around 55 °C, while the increase for the oxidation components appears to take place also around 55 °C, showing continuous increasing trend for the whole tested temperature range. This is in accordance with other related studies [13,21], where extraction temperatures above 55 °C were not considered and were not studied as a means to avoid thermal volatile alterations and oxidative degradation of the olive oil.

Regarding DOE-RSM optimization, a quadratic model including all linear terms, two-way interaction terms and second-order terms was fitted to the response data. The Multiple R-squared, Adjusted R-squared, *p*-value, and output of the Analysis of Variance (ANOVA) table for the model can be seen in Table 1.

As indicated by the Multiple R-squared, Adjusted R-squared, and *p*-value, the quadratic model is a good fit to the data and is statistically significant. There is also no evidence of lack of fit in the model. By studying Table S1, one can see that the most significant terms are extraction temperature and extraction time. The interaction between desorption temperature and desorption time also appears to be of significance.

**Figure 2:**
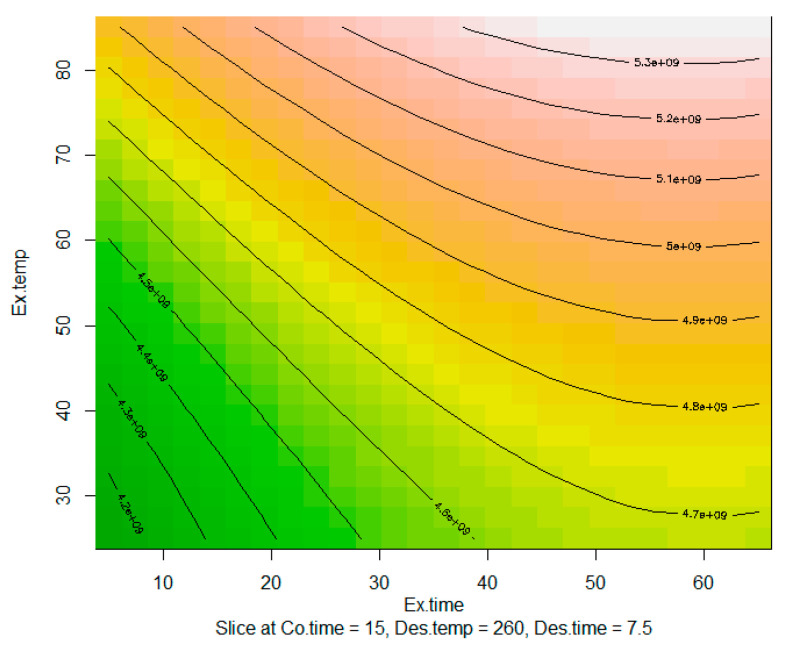
Contour plot for factors extraction temperature (Ex.temp) and extraction time (Ex.time). Green colour indicates lower response values and pink colour higher response values. Contours include corresponding response levels. The contour plot is a slice at the centre points for the rest of the remaining factors (Conditioning time (Co.time), Desorption temperature (Des.temp), and Desorption time (Des.time)). (e + 08 = × 10^8^).

A contour plot for the response surface of extraction time and extraction temperature can be seen in Figure 2. The plot indicates that the optimal point for these two factors is close to their maximum values. It also indicates that extraction time matters less as extraction temperature increases and vice-versa. For example, at a temperature of 85 °C, the same effect is achieved with an extraction time between 40 and 65 min. However, the initial screening study indicated that at temperatures above 55 °C an increase in oxidation products is witnessed. Therefore, a safer factor level selection would be an extraction temperature of 55 °C and an extraction time of 50 min.

As the interaction between desorption time and desorption temperature appears significant, by studying their contour plot at Figure S1 at the selected extraction time and temperature, one can see a saddle-like plot, which suggests that optima lie with one factor maxed or the other. To keep desorption temperature as low as possible to avoid any oxidation or thermal degradation effects at the inlet, a selection of desorption temperature of 240 °C and desorption time of 12 min is made.

Regarding conditioning time, all the models’ terms for this factor appear not to be significant, so a choice of the shortest time is made, in this case 5 min. In summary, the SPME conditions chosen for analysis are 55 °C of extraction temperature, 5 min of conditioning time, 50 min of extraction time, and 240 °C and 12 min desorption temperature and time, respectively.

**Figure 3:**
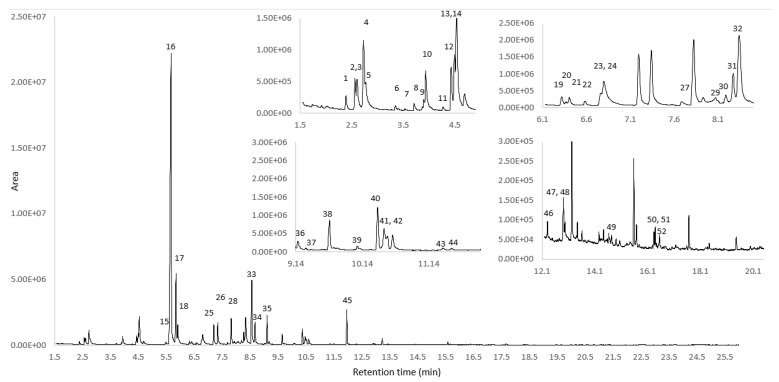
Major VOCs identified in the TIC of a QC olive oil sample analysed with optimised HS-SPME/GC-MS conditions. Upper in the insets, zoomed areas are shown. The identified components are given in numbers. (1) 1-methoxy-2-propanol, (2) 1-penten-3-ol, (3) 1-penten-3-one (4) 3-pentanone, (5) pentanal, (6) 3-methylbutan-1-ol, (7) (*E*)-2-pentenal, (8) (*E*)-2-pentenal, (9) 1-pentanol, (10) 2-penten-1-ol, (*Z*)-, (11) 1-octene, (12) octane (13) 3-hexenal, (14) hexanal, (15) (*Z*)-hex-3-en-1-ol, (16) (2*E*)-hexenal, (17) (*E*)-2-hexen-1-ol, (18) 1-hexanol, (19) (prop-2-en-1-yl)cyclopentane, (20) o-xylene, (21) 3-ethyl-1,5-octadiene, (22) heptanal, (23) (*Z*)- 2-penten-1-ol, acetate, (24) 2,4-hexadienal, (25) 3-ethyl-1,5-octadiene, (26) 3-ethyl-1,5-octadiene, (27) 2,2-dimethyl-3-heptanone, (28) 5-ethyl-2(5*H*)-furanone, (29) hexanoic acid, (30) 6-methyl-5-hepten-2-one, (31) 3-ethyl-1,5-octadiene, (32) 3-ethyl-1,5-octadiene, (33) (3*Z*)-hex-3-en-1-yl acetate, (34) hexyl acetate, (35) eucalyptol (I.S), (36) benzyl alcohol, (37) o-cymene, (38) 2,2-dimethyl-3-heptanone, (39) 1-undecene, (40) nonanal, (41) (*E*)-4,8-dimethyl-1,3,7-nonatriene, (42) 2-ethylhexanoic acid, (43) octanoic acid, (44) 1-nonanol, (45) (*E*)-2-dodecene, (46) 11-hexadecen-1-ol, (*Z*)-, (47) nonanoic acid, (48) 1-dodecene, (49) copaene, (50) pentadecane, (51) eremophilene, (52) alpha-farnesene. (E + 07 = × 10^7^).

The aim of the present study was the development of a method for the comprehensive profiling of the volatile content of VOOs. The oven program seemed to be appropriate for the most volatile components, since at 17 min and 180 °C all components of interest were eluted. The DVB/CAR/PDMS fiber showed high extraction efficiency and was selected for further study. Extraction temperature and extraction time had a great effect on the detection of volatile components and their composition. With an extraction temperature of 55 °C and an extraction time of 50 min, an increased area of the total ion chromatogram was observed, preventing thermal oxidation or degradation of the compounds of interest. The optimum HS-SPME/GC-MS conditions were applied in the profiling method, facilitating the identification and semi-quantitation of 92 VOCs of different chemical classes, as shown in Table 2. A representative gas chromatogram of a Quality Control (QC) sample analysed with the optimal HS-SPME/GC-MS conditions is shown in Figure 3.

### 2.2. Identification of VOCs by HS-SPME/GC-MS Analysis 

Olive oil volatile compounds are primarily derived from the enzymatic oxidation of the fatty acids linoleic and linolenic. Positive aroma perceptions in olive oil are produced by endogenous plant enzymes, through the LOX, while aroma defects are linked with chemical oxidation and exogenous enzymes [5]. The volatile fraction of VOOs mainly consists of aldehydes, ketones, aliphatic and aromatic hydrocarbons, ethers, esters, aliphatic and triterpenic alcohols, and furan derivatives. Aroma compounds responsible for green desirable notes, found in high-quality VOOs, are produced enzymatically from polyunsaturated fatty acids through the LOX pathway. It has been observed that in the aroma of these oils, C6 aldehydes and alcohols and their corresponding esters are the most abundant accumulation products [26]. Both major and minor VOCs are important to olive oil aroma, since minor compounds with low odor threshold values are frequently considered significant contributors. Even those whose levels are below their olfactory threshold can still provide useful quality marker information [27].

**Figure 4:**
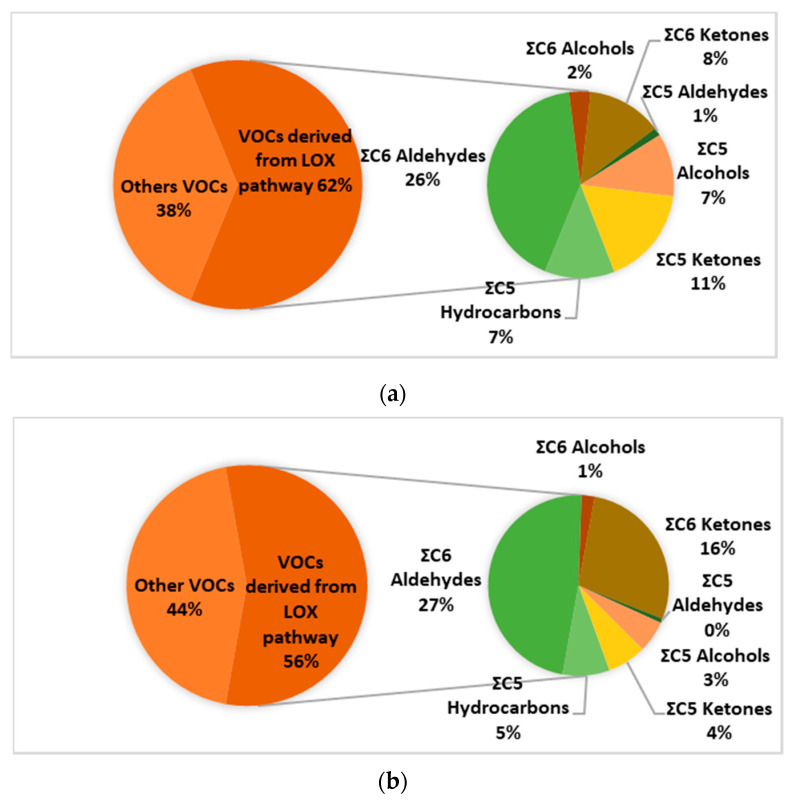
Natural variation of VOCs derived from LOX pathway in a (**a**) Koroneiki and (**b**) Tsounati variety sample.

In all samples, C6 and C5 VOCs derived from the LOX pathway were the most abundant (Figure 4a,b). This is in accordance with the organoleptic results obtained by the tasting panel, where samples were identified as EVOOs with medium fruitiness attributes (3 < x < 6). C6 and C5 VOCs contribute to the delicate green aroma of VOO, which enhances the “green” fruitiness attribute and is highly valued by consumers. The formation of these compounds is mainly influenced by the cultivar, the degree of ripeness of the fruit, the storage period of fruits before oil extraction, and the processing method [28].

C6 aldehydes produced from the linolenic acid, *E*-2-hexanal, 3-hexenal, and *Z*-2-hexanal, were found in considerable amounts. In agreement with data reported in other studies examining European olive oil cultivars, (2*E*)-hexenal, responsible for the “green” attributes, was the most prevalent aroma compound in all VOO samples [20]. More specifically, concerning the Koroneiki cultivar, (2*E*)-hexenal ranged from 7.64 mg kg^−1^ in a conventional sample to 51.25 mg kg^−1^ in an organic sample from the Heraklion region unit. In a similar manner, *Z*-3-hexanal, which is positively correlated with fruity, leave/grass, and almond notes [29], was found in high levels in VOO samples, reaching 8.77 mg kg^−1^ in an organic sample from Chania. High amounts of hexanal, produced by breaking 13-hydroperoxide from linoleic acid, are frequently related to sensory notes sweet, apple, and green [30]. In the studied samples, hexanal reached 8.04 mg kg^−1^ in an organic sample. C6 alcohols such as *Z*-3-hexen-1-ol, *E*-2-hexen-1-ol, and hexan-1-ol, related to sweetness, were also identified [5]. *Z*-3-hexen-1-ol and hexan-1-ol were then esterified by the alcohol acetyltransferase to produce the corresponding esters (3*Z*)-hex-3-en-1-yl acetate and hexyl acetate, which also were found in considerable amounts in the studied samples ranging from 2.13 to 40.08 mg kg^−1^ and from 0.5 to 9.75 mg kg^−1^, respectively. The contribution of these volatiles is quite significant, as they complement aromas with sweet and pleasant notes [31].

Aside from C6 molecules, the headspace of VOO contains a reasonable amount of C5 alcohols and C5 carbonyl compounds [32]. The existence of these VOCs indicates the development of a new branch of the LOX pathway that produces C5 compounds. This extra branch is active during the biogeneration of olive oil aroma. C5 aldehydes and alcohols contribute to the positive flavor attributes of olive oil, providing pungent sensations, and are correlated to bitterness [5]. The molecule 1-penten-3-ol is related to “mouldy” and “rancid” defects associated with lawn, olive, leaf, and pungency and ranged from 0.90 mg kg^−1^ in conventional VOO of Heraklion to 2.94 mg kg^−1^ in an organic sample of Koroneiki cultivar in Chania. (*Z*)-2-penten-1-ol (0.60–1.87 mg kg^−1^) and 2-pentenal (*E*)- (0.1–0.51 mg kg^−1^) are minor compounds whose presence is correlated with banana, sweet, fruity and green, apple, and bitter almond attributes, respectively [33]. C5 ketones, pentene dimers, or monoterpenes affect olive oil aroma even in low concentrations. The molecule 1-penten-3-one reached high amounts at the Koroneiki samples of Heraklion (5.75 mg kg^−1^) whereas it was not detected in an organic VOO of the Koroneiki variety from Rethymnon. Its presence is linked to leaf, mustard, and pungent notes [34], whereas higher amounts have been also related to metallic notes. The isomer 3-ethyl-1,5-octadiene is produced over the oil extraction process through the same branch of the LOX pathway as the C5 compounds and was detected in all samples (0.12–4.91%) [35]. Other minor volatiles, terpene hydrocarbons (mono- and sesquiterpenes), were detected in Cretan VOOs, and despite the fact they do not contribute to VOO aroma due to their low concentration and high odor threshold, they constitute significant quality markers [29]. Among all terpenoid compounds, the most abundant in the studied VOO samples, were d-limonene (0.04–20.18 mg kg^−1^) with citrus, mint odor perceptions, α-farnesene (0.02–0.27 mg kg^−1^) with floral, herb, wood, and sweet odor qualities [36], and (*E*)-4,8-dimethyl-1,3,7-nonatriene (0.04–0.19 mg kg^−1^). Other terpenoid volatiles such as alpha- and beta-pinene, o-cymene, and copaene were also detected in lower amounts. Some rare terpenic compounds such as citral, (+)-cyclosativene, eremophilene, neral, 3-carene, and kessane, which so far have been reported only in a few studies [29,37,38], were also identified with the current method.

The detection of benzenes, toluene, xylenes, and styrene (BTEXS) in olive oil is of high importance. These VOCs are widely distributed in the environment and food products mainly due to emissions from vehicles, bonfires, and paints into the ambient air near orchards. In olive oil, BTEXS are frequently identified, and their presence is attributed to several factors, such as biological processes in the fruit, the production technology used, contamination by fuel vapors, etc. [39]. In this study, toluene, styrene, xylene, propylbenzene, 1-ethyl-2-methylbenzene, 4-ethyltoluene, 1,4-diethylbenzene, and naphthalene were identified. The presence of styrene in VOO samples has been attributed to natural causes, such as decarboxylation of the cinnamic acid that is naturally present in the olive pulp or by migration from the plastic package [39]. Naphthalene is usually hanged on trees by olive farmers, as it has a characteristic aroma that is theorised to repel the olive fly (Bactrocera oleae), and in this study was identified in low levels (0.01–0.92 mg kg^−1^) in 8 out of 63 samples [40].

According to our knowledge, VOCs such as 1-propanone-1-phenyl, benzene, 1,4-diethyl, 11-hexadecen-1-ol, (*Z*), and 2,4-di-tert-butylphenol are reported for the first time in VOO samples. 

### 2.3. Classification of Cretan VOO Samples 

EVOO flavor is considered a significant quality but may also be utilized as a classification criterion. VOO volatile profile plays a fundamental role in defining the geographical origin of oils. The majority (roughly 70%) of PDO/PGI olive oils in Greece are produced in the Peloponnese and Crete. Six of the ten Cretan PDOs come exclusively from Koroneiki, the main Greek cultivar and also the most prominent in Crete; three include blends of 90% Koroneiki VOO, and only one contains blends of 60% oil from Tsounati, the second most significant Cretan cultivar [41]. One PDO was registered for the entire Lasithi regional unit, whilst two PDOs from Chania come from two distinct terroirs but include comparable blends of Koroneiki and Tsounati oils. It is essential that these specific PDOs be subjected to thorough administrative control. These objective criteria, including organoleptic ones, should be accessible to support the authenticity and prevent attempts at fraud. 

In the current study, the potential of the newly developed method was investigated for its use in the classification of Cretan VOO samples with the aid of multivariate statistical analysis. The obtained data were analysed using multivariate statistical analysis models to identify correlations between VOC profiles and VOO geographical origin; thus, chromatographic peak areas were used for modelling the differences between the studied groups. The overall performance of the analytical method was evaluated by assessing the variability of the detected compound in QC samples. All the compounds in QC samples presented % RSD < 30%, indicating satisfactory stability and reproducibility of the analytical system during the analytical batch. As shown in Figure S2, principal component analysis (PCA) was used to evaluate the analytical system’s suitability and reproducibility (QC cluster). Group differentiation and identification of potential markers related to geographical origin were performed by latent structures OPLS DA analysis. 

**Figure 5:**
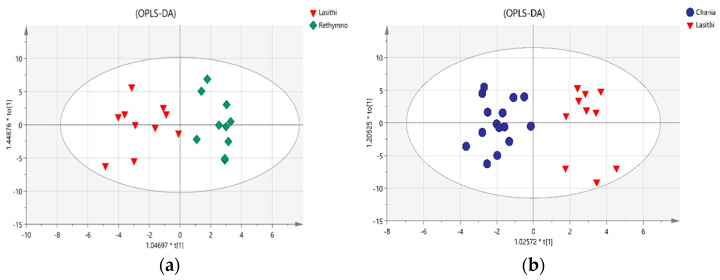
Orthogonal Projections to Latent Structures Discriminant Analysis (OPLS-DA) on EVOO samples volatile profile, using the ‘geographical origin’ as class membership criterion, (**a**) Lasithi vs. Rethymno, (**b**) Chania vs. Lasithi.

**Figure 6:**
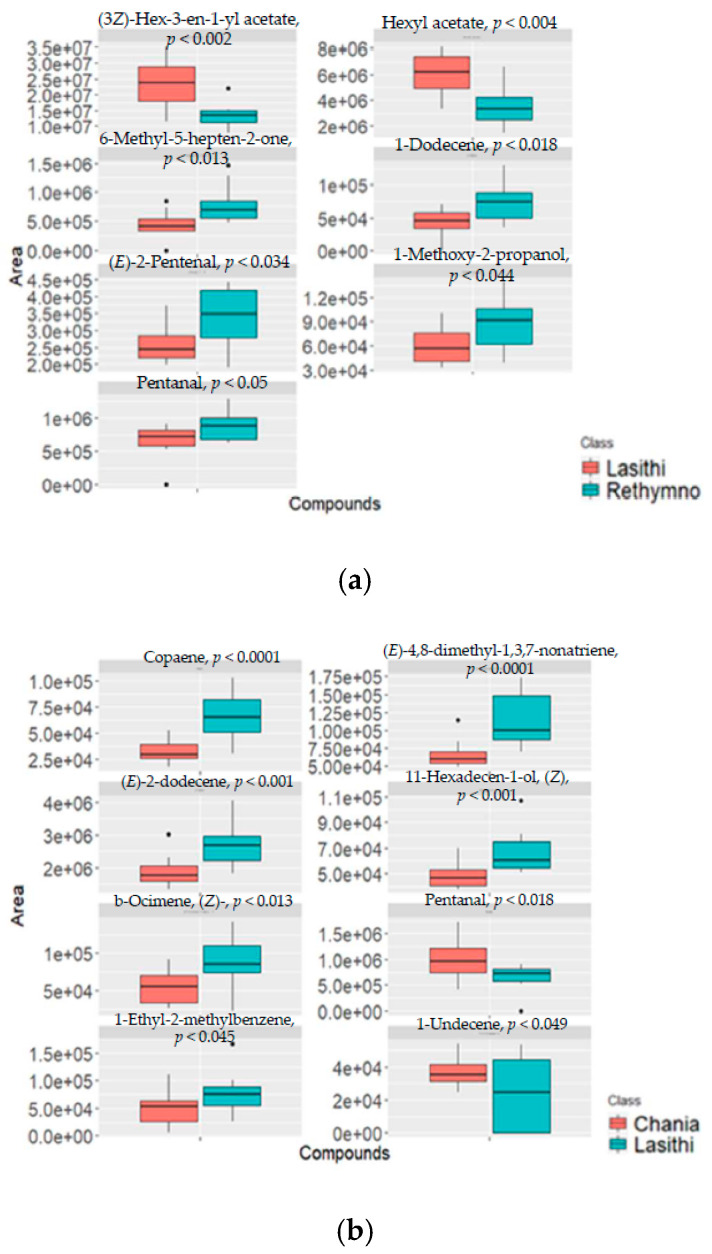
Box plots of identified biomarkers in VOOs samples. Boxes are drawn from the 25th to 75th percentiles in the concentration distribution. (**a**) Lasithi vs. Rethymnon, (**b**) Chania vs. Lasithi. (e + 07 = × 10^7^).

Before the assessment of geographical origin to the volatile fingerprint of VOOs, the effect of farming type (organic vs. conventional) was examined. Multivariate statistical analysis was performed to samples from each area produced with different cultivation types. It was observed that farming type is not a statistically significant factor for the specific samples, and thus geographical origin assessment was further performed using both sample types (organic and conventional). Initially, VOO samples from nearby production regions such as Chania-Rethymnon, Rethymnon-Heraklion and Lasithi-Heraklion (Figure S3) did not present any statistically significant differences regarding their volatile content. Although the region of Chania is located on the western side of Crete and Heraklion roughly in the middle of the island, the content of VOOs was not affected by the different origins due probably to other stronger common factors that may affect the VOO profile, which may include climate, fruit maturity, and processing technology [5]. The impact of geographical origin was found significant when VOOs from Lasithi, the easternmost region of Crete, were compared with samples from Chania and Rethymnon. The respective orthogonal projection to latent structures discriminant analysis (OPLS-DA) models are illustrated in Figure 5a,b, where samples with the same origin are clustered together and show clear differentiation against samples from Lasithi. Clear separation was observed between VOOs from Chania and Lasithi and between VOOs from Lasithi and Rethymnon with cross-validated ANOVA analysis (CV Analysis), *p* = 0.0008 and *p* = 0.02, respectively. Permutation tests showed that the models were robust, with high predictability (R2Y (cum) and Q2 values of 0.827 and 0.656 and 0.881 and 0.453, respectively). Studying the multivariate loadings vectors in each case revealed various analytes related to the differentiation between the studied groups. As a final step, the unpaired Student’s t-test was performed to establish and confirm the significance of important metabolites. All statistically significant metabolites and their variations in each group are presented as box plots in Figure 6.

The compounds found significant (*p* < 0.05) for geographical differentiation consisted of four terpenic hydrocarbons (6-methyl-5-hepten-2-one, copaene, (*E*)-4,8-dimethyl-1,3,7-nonatriene, and (*Z*)-beta-ocimene), two esters ((3*Z*)-hex-3-en-1-yl acetate and hexyl acetate), two aldehydes ((*E*)-2-pentenal and pentanal), three hydrocarbons (dodecene, (*E*)-2-dodecene, undecene, and 1-ethyl-2-methylbenzene), one alcohol ((*Z*)-11-hexadecen-1-ol), and one ether (1-methoxy-2-propanol), as shown in Figure 6. Eight of them, 6-methyl-5-hepten-2-one, copaene, (*E*)-4,8-dimethyl-1,3,7-nonatriene, (*Z*)-beta-ocimene, (3*Z*)-hex-3-en-1-yl acetate, hexyl acetate, (*E*)-2-pentenal, and dodecene, have previously been reported as markers for the classification of Greek VOO according to geographical origin [42,43]. More specifically, efforts have been put on studies that focus on the differentiation of olive oils produced in different islands of north-western Greece [43] (Zakynthos, Kefalonia, Lefkada, Kerkyra, Preveza, and Messologi) and different regions [42] (Messinia and Lakonia in the Peloponnese, Irakleio on the island of Crete, and Etoloakarnania in central Greece).

In this study, when samples from Lasithi and Rethymnon were compared, seven volatiles were found to be responsible for the differentiation between the groups. Three of them ((3*Z*)-hex-3-en-1-yl acetate, hexyl acetate and (*E*)-2-pentenal) are produced through the LOX-pathway, contributing to olive oil aroma. The volatiles 1-methoxy-2-propanol and 1-dodecene have also been identified as potential markers in Croatian monovarietal/PDO EVOO [44], while pentanal has shown high discrimination ability for the classification of north Moroccan olive oils [45]. A ripening indicator with fruity-like odor, 6-Methyl-5-hepten-2-one, has been detected as a marker for the characterisation of Turkish olive oils according to origin (Mediterranean, Aegean, South-eastern Anatolia, Marmara, and Black Sea) [46]. Overall, (3*Z*)-hex-3-en-1-yl acetate, and hexyl acetate were found upregulated in samples from Lasithi while all the others were increased in samples from Rethymnon. Pentanal was found as statistically significant in both origin studies of VOOs. Samples from Lasithi showed lower levels of pentanal compared to Rethymnon and Chania.

In total, eight compounds were found to differ between VOOs from Chania and Lasithi. Three terpenic hydrocarbons, copaene, with a sweet-fruity description, (*E*)-4,8-dimethyl-1,3,7-nonatriene, and (*Z*)-beta-ocimene, with an herb aroma description, were among the most important discriminant volatiles. This is in accordance with several studies where terpene hydrocarbons were reported as suitable markers of the geographic origin and genotype of EVOOs [29,47,48]. (*E*)-2-Dodecene has been reported as a discriminant variable in a study investigating VOOs of Chemlali and Neb Jmel cultivars growing in central and south Tunisia [49]. In this study, 1-ethyl-2-methylbenzene, 1-undecene, and (*Z*)-11-hexadecen-1-ol are reported as discriminant volatiles for geographical origin classification for the first time. In general, pentanal and 1-undecene, were found in increased amounts in oils from Chania, while the other statistically significant compounds were found in decreased levels in oils from Lasithi. 

The aroma profile of the monovarietal Koroneiki VOOs examined was mainly characterized by the presence of C6 LOX alcohols, aldehydes, and esters as well as the contribution of C5 LOX compounds similarly to literature reports for VOOs of the same cultivar [6,50]. VOCs concentrations in the monovarietal Koroneiki VOOs of the current study varied significantly either as total content, or as total group content, or even as content of most individual VOCs. Differences were considered significant even within the same farming type (organic or conventional) or production region, revealing the complexity of the VOO aroma profile. The latter is in accordance with reported findings on variable profiles expected due to the effect and synergism of various biotic and abiotic parameters, such as agricultural practices, altitude, harvest time, and conditions employed from harvest-to-oil production and storage [51]. Nonetheless, VOCs identified in the current study as positive contributors (e.g., (2*E*)-hexenal, hexanal, hexan-1-ol, 1-penten-3-one, hexyl acetate, and hexenyl acetate), in terms of amount and/or importance of contribution to overall profile (relevant to OTV), have been also recognized as such for monovarietal Koroneiki VOOs studied [6,50]. Moreover, terpenic compounds useful indicators for geographical and botanical VOO differentiation have been reported to be present in the aroma profile of Koroneiki VOOs [6,50], which is in line with current findings; limonene, copaene, and a farnesene were present in all monovarietal Koroneiki VOO samples studied.

The application potential of the method could be improved by analysing a larger dataset of EVOO samples and/or samples from oncoming harvest years from the same areas. We are planning to expand our data set in this direction, analysing samples produced in subsequent harvest years. In addition, we plan to use this technology to compare Cretan vs. non-Cretan VOOs, a comparison that could be of legislating and commercial significance.

## 3. Materials and Methods

### 3.1. Samples

A total of 63 bottled and branded VOO samples that were previously evaluated for quality parameters were collected for the 5th Cretan olive oil competition. The samples were produced from olives of both organic (n = 19) and conventional (n = 44) olive groves and different local olive cultivars (Koroneiki and Tsounati) harvested in 2018–2019 in different regions of Crete (Chania n = 15, Rethymnon n = 12, Lasithi n = 10, and Heraklion n = 26) (Table S2). The organoleptic assessment of the olive oil samples was performed according to the Official EU method EEC/2568/91 [4] and its amendments by the trained sensory panel of the accredited Sensory Evaluation Laboratory of Crete ACR in Rethymnon Crete, Greece, and stored at −20 °C in dark glass vials that were shielded from light and oxygen.

### 3.2. Chemicals

LC-MS grade methanol was purchased from Sigma-Aldrich (Darmstadt, Germany). The internal standard eucalyptol (1,8-cineole) was of analytical grade and was purchased from Sigma-Aldrich (Darmstadt, Germany).

### 3.3. Optimisation of SPME Extraction Conditions

Initially, the effect of 3 experimental conditions for the HS-SPME method was studied by triplicate analysis following the well-established one-factor-at-a-time approach (OFAT). Sample mass was studied in the range of (1.000–4.000 g) in order to investigate any fiber saturation effects. Additionally, a range of settings was investigated for stirring of the sample (100–300 rpm). The latter two were optimised using the total peak area. The effects of extraction temperature for VOCs that are both desirable and undesirable were screened in the range of 30 to 85 °C so that an initial assessment was made for this crucial experimental parameter. Regarding the optimisation of the rest SPME conditions, a modelling approach was performed using the DOE and RSM approaches for the total peak area of each chromatogram representing all the ionisable and detectable volatile components of the sample. The optimisation was performed using a base fractional factorial (FF) 2^5−1^ design with 5 factors (resolution V) that was augmented to a circumscribed central composite (CCC) with an alpha value of 2. A total of 32 runs included 16 base runs for FF (cube portion), 6 centre points to measure the experimental error, and 10 runs for CCC (star portion). Runs were randomised for each portion and were run in a single analytical sequence. The SPME factors selected for optimisation were extraction temperature, conditioning time, extraction time, desorption temperature, and desorption time. Although a preliminary screened factor, extraction temperature was included in the model as a significant factor to have a more complete picture of the SPME condition effects and to increase the model’s applicability. The experimental area for the corresponding factors is given in Table 3, with the corresponding design provided in Table S3. Factor and level selection was made based upon instrumental limitations, fit-for-purpose needs, and by referring to relevant literature [18,52].

The response selected for optimisation was the total chromatogram area, as measured by the sum of the areas of each ion, after the removal of the ions that were attributed mainly to siloxane contamination, as described in Section 3.6.

### 3.4. Sample Preparation

The optimal SPME conditions were set following the approach described in Section 3.3. Briefly, 1.85 gr of olive oil were weighed into a 15 mL glass vial, and 4 μL of a 1000 μg/mL eucalyptol internal standard solution in methanol were added, reaching a final concentration of 2 μg/mL. The vials were closed with a PTFE/silicone septum and equilibration was performed with a PAL Shimadzu autosampler unit (AOC 6000, CTC Analytics, Zwingen, Switzerland) at 55 ± 0.1 °C for 15 min upon agitation at 250 rpm. The (DVB/CAR/PDMS) fiber (2 cm length, 50/30 thickness) (Sigma-Aldrich) was then introduced to the headspace for 50 min at 55 °C. The fiber was pre- and post-conditioned for 10 min at 260 °C, according to the manufacturer’s instructions.

### 3.5. Gas Chromatography-Mass Spectrometry

VOCs were analyzed using a Shimadzu GCMS-QP2020 instrument equipped with a PAL SHIMADZU autosampler unit (AOC 6000, CTC Analytics, Zwingen, Switzerland). Chromatographic separation was performed on a MEGA-5 MS capillary column (30 m × 0.25 mm, 0.25 μm) (MEGA, Legano, Italy). Injection was operated in split mode (1:2) at 250 °C; He as the carrier gas at a flow rate of 1.2 mL/min; the column was held for 2.5 min at 40 °C, then programmed at 10 °C min^−1^ to 230 °C and held for 5 min. The mass detector was operated in the electron impact mode at 70 eV. Temperatures of MS source and quadrupole were set to 240 and 200 °C, respectively. The mass spectra scanned at *m*/*z* 40–450 amu range.

A pooled sample (QC) was prepared as a representative sample by mixing equal volumes of each VOO sample. The QC sample was injected at the start, every seven samples, and the end of the run to evaluate instrument stability. Blank runs were performed during the study to reveal possible carryover. The samples were analysed in randomised order.

### 3.6. Data Processing and Chemometrics

The Shimadzu software LabSolutions, GCMS Solutions version 2.50 SU3LabSolution, was used for acquiring the GC-MS data. Assignment Validator and Integrator (GAVIN) script for MATlab was used for performing peak integration complementary to Automated Mass spectral Deconvolution and Identification System (AMDIS) for peak deconvolution and identification. Total peaks areas for RSM-related modeling were extracted from the raw chromatogram files using OpenChrom^®^ Lablicate Edition version 1.4 (Lablicate GmbH, Hamburg, Germany) [53] including the area of all scanned ions minus those attributed to siloxane contamination from column: 207, 281, and 355 [54]. Compound identification was performed by comparing mass spectra of eluting compounds with those of commercial libraries (NIST17 and FFNSC3) and by calculating linear retention indices relative to a series of n-alkanes (C8-C24) (Sigma-Aldrich). The VOCs were semi-quantified by dividing the peak areas of the compounds of interest by the peak area of the internal standard (Eucalyptol) and multiplying this ratio by the initial concentration of the internal standard (expressed as mg kg^−1^). The peak areas were measured from the full scan chromatograph using total ion current (TIC). 

DOE design and analysis were performed using R version 3.6.2 [55] with RStudio (RStudio, Inc., Boston, MA, USA) [56] and DOE/RSM-related functionality [57,58,59]. The SIMCA package (version 13.0.2.; Umetrics, Sweden) was used for multivariate statistical analysis and biomarker assessment via VIP plots (Variable Importance for the Projection), loading plots, S-plots, p (corr), and hotelling’s lines. Principal components analysis (PCA) and orthogonal projection to latent structures discriminant analysis (OPLS-DA) were performed to assess data in a multivariate setting. Models’ validation was evaluated using permutation plots and CV-ANOVA value. Two-tailed t-test, with unequal variance and a threshold of *p* < 0.05, and ANOVA were employed in Microsoft Excel Spreadsheets.

## 4. Conclusions

In this study, a HS-SPME/GC-MS method by Design of Experiments (DoE) and Response Surface Methodology (RSM) approaches was successfully optimized. The optimized parameters were sample amount, sample stirring, extraction temperature, conditioning time, extraction time, desorption temperature, and desorption time. The optimized HS-SPME/GC-MS method allowed the identification and semi-quantification of a high number of compounds. Of the 92 VOCs identified, 4 are found for the first time in EVOO while others, mainly terpenoid compounds, are rarely identified in EVOO, with the use of SPME/GC-MS. Statistical analysis allowed for identifying markers with significance in the geographical classification of Cretan EVOO samples. The method is deemed useful for the analysis of EVOO, providing easy, automated, efficient, and economic sample preparation and analysis along with information-rich GC-MS data.

## Figures and Tables

**Table 1:** Model fit data along with corresponding output from the ANOVA analysis table.

**Multiple R-Squared**	0.9028				
**Adjusted R-Squared**	0.7262				
***p*** **-Value**	4.02 × 10^−3^				
	**Df**	**Sum Sq**	**Mean Sq**	***F *** **Value**	**Pr (>F)**
**FO (x1, x2, x3, x4, x5)**	5	1.2055 × 10^18^	2.4111 × 10^17^	16.3748	9.02 × 10^−5^
**TWI (x1, x2, x3, x4, x5)**	10	1.9382 × 10^17^	1.9382 × 10^16^	1.3164	0.3283
**PQ (x1, x2, x3, x4, x5)**	5	1.0584 × 10^17^	2.1168 × 10^16^	1.4376	0.2855
**Residuals**	11	1.6197 × 10^17^	1.4724 × 10^16^		
**Lack of Fit**	6	4.5182 × 10^16^	7.5303 × 10^15^	0.3224	0.8996
**Pure Error**	5	1.1679 × 10^17^	2.3357 × 10^16^		

FO stands for First Order terms, TWI for Two Way Interaction terms and PQ for Pure Quadratic terms. Df are the Degrees of Freedom, Sum Sq the Sum of Squares and Mean Sq the Mean Square. Extraction temperature (x1), conditioning time (x2), extraction time (x3), desorption time (x4) and desorption temperature (x5).

**Table 2:** Chromatographic data (retention time, retention index) and mean values (mg/kg) relative to internal standard (Eucalyptol) and standard deviation for identified volatile compounds in Cretan EVOO samples.

Compound Name	Rt	RIexp	RI Lit	Chania Mean (n = 17)	±SD	Heraklion Mean (n = 26)	±SD	Lasithi Mean (n = 10)	±SD	Rethymnon Mean (n = 10)	±SD
**Alcohols**											
Isobutanol	2	626	622	0.06	0.05	0.06	0.05	0.1	0.04	0.07	0.06
1-Penten-3-ol, C5	2.54	683	686	1.96	0.38	1.69	0.49	1.45	0.49	1.05	0.37
3-Methylbutan-1-ol	3.33	738	744	0.12	0.05	0.14	0.05	0.15	0.05	0.13	0.07
1-Pentanol	3.89	771	766	0.09	0.08	0.08	0.06	0.04	0.05	0.17	0.05
(*Z*)-2-Penten-1-ol, C5	3.92	774	767	1.39	0.25	1.05	0.36	0.86	0.35	0.85	0.19
(*Z*)-Hex-3-en-1-ol, C6-LnA	5.53	857	858	0.12	0.04	0.12	0.05	0.09	0.05	0.19	0.06
(*E*)-Hex-2-en-1-ol, C6-LnA	5.82	871	866	1.99	1.75	2.23	2.49	0.72	2.36	7.96	3.45
Hexan-1-ol, C6-LA	5.9	875	862	1.77	0.9	1.62	0.88	0.99	0.9	4.31	1.12
1-Octanol	9.76	1076	1074	0.08	0.02	0.08	0.03	0.07	0.02	0.07	0.04
1-Nonanol	11.48	1176	1172	0.06	0.03	0.1	0.05	0.1	0.04	0.05	0.04
(*Z*)-11-Hexadecen-1-ol	12.31	1228	-	0.05	0.01	0.05	0.02	0.05	0.02	0.03	0.02
**Aldehydes**											
3-Methylbutanal	2.26	652	650	0.04	0.03	0.04	0.04	0.05	0.03	0.05	0.03
(*E*)-2-Pentenal, C5	3.52	749	754	0.08	0.03	0.06	0.03	0.06	0.02	0.04	0.02
(*E*)-2-Pentenal, C5	3.69	760	754	0.3	0.08	0.25	0.09	0.22	0.09	0.17	0.07
Pentanal	2.75	703	695	0.99	0.44	0.87	0.4	0.75	0.39	0.37	0.22
3-Hexenal, C6-LnA	4.48	806	795	2.85	2.78	1.39	1.85	0.83	1.66	1.84	0.87
Hexanal, C6-LA	4.52	808	798	3.51	1.42	3.1	1.06	2.75	1.07	4.22	1.08
2-Hexenal, C6-LnA	5.47	854	854	0.19	0.05	0.16	0.05	0.15	0.05	0.22	0.07
(2*E*)-Hexenal, C6-LnA	5.61	862	856	25.43	9.62	22.16	9.57	21.51	9.23	32.67	13.59
Heptanal	6.6	908	903	0.19	0.07	0.21	0.07	0.17	0.06	0.12	0.06
2,4 Hexadienal	6.79	919	916	1.95	0.71	1.43	0.61	1.22	0.57	1.66	0.34
Benzeneacetaldehyde	9.39	1055	1049	0.03	0.01	0.04	0.02	0.06	0.02	0.06	0.02
Nonanal	10.38	1111	1099	0.98	0.47	1.14	0.54	1.17	0.5	0.45	0.47
**Acids**											
Acetic acid	1.72	596	595	0.56	0.56	0.52	0.56	0.34	0.49	0.57	0.89
3-Methylbutanoic acid	5.35	852	858	0.02	0.01	0.02	0.01	0.02	0.01	0.03	0.01
Hexanoic acid	8.04	981	981	0.73	0.71	0.55	0.51	0.21	0.49	0.7	0.35
Heptanoic acid	9.72	1074	1076	0.1	0.03	0.09	0.03	0.06	0.03	0.09	0.03
2-Ethylhexanoic acid	10.52	1118	1123	0.58	0.55	0.37	0.45	0	0.41	0.37	0.41
Octanoic acid	11.36	1170	1177	0.14	0.06	0.13	0.05	0.06	0.05	0.14	0.04
Nonanoic acid	12.92	1268	1270	0.25	0.1	0.25	0.1	0.1	0.09	0.26	0.09
**Esters**											
Ethyl acetate	1.89	615	614	0.22	0.12	0.27	0.13	0.29	0.12	0.22	0.16
3-Methylbutyl acetate	6.04	882	883	0.13	0.08	0.13	0.07	0.18	0.07	0	0.11
(*Z*)-Pent-2-en-1-yl acetate	6.76	918	916	0.59	0.33	0.39	0.25	0.27	0.23	0.51	0.11
(3*Z*)-Hex-3-en-1-yl acetate, C6-LnA	8.55	1009	1005	18.49	9.03	12.15	7.19	13.46	7.22	7.97	4.09
Hexyl acetate, C6-LA	8.68	1016	1011	4.5	2.44	3.07	1.85	4.21	1.89	2.81	1.51
**Ethers**											
1-Methoxy-2-propanol	2.36	654	650	0.08	0.03	0.11	0.08	0.1	0.07	0.08	0.03
1-Methoxyhexane	4.99	830	831	0	0	0	0.02	0	0.06	0	0
(3*Z*)-1-Methoxy-3-hexene	5.05	838	832	0	0	0	0	0	0.08	0	0
**Ketones**											
1-Penten-3-one, C5	2.58	687	679	1.96	0.92	1.65	1.17	1.46	1.07	0.7	1.15
3-Pentanone	2.71	701	694	1.65	0.92	1.26	0.72	1.04	0.75	1.51	0.77
2,2-Dimethyl-3-heptanone	9.64	1070	-	5.07	5.21	2.07	3.54	0.85	3.14	3.22	1.13
**Terpenic Compounds**											
.alpha.-Pinene	7	930	932	0.01	0.04	0.02	0.07	0	0.06	0.18	0.03
.beta.-Pinene	8.05	983	980	0.07	0.26	0.07	0.31	0	0.27	0	0.15
6-Methyl-5-hepten-2-one	8.2	990	985	0.61	0.41	0.56	0.33	0.51	0.31	0.78	0.34
Alpha-Terpinene	8.79	1018	1017	0	0	0	0.01	0	0.01	0.02	0
3-Carene	8.79	1018	1018	0	0.01	0.01	0.01	0	0.01	0.02	0
(*Z*)-beta-Ocimene	9.29	1050	1048	0.05	0.02	0.07	0.03	0.07	0.03	0.09	0.02
o-Cymene	8.95	1031	1037	0.14	0.48	0.2	0.59	0	0.54	1.33	0
d-Limonene	9.02	1035	1041	0.7	1.83	0.99	3.29	0.1	2.87	0.19	4.33
(+)-2-Carene	9.04	1035	1031	0	0	0.06	0.23	0	0.23	0.92	0
Neral	12.61	1247	1244	0	0.01	0	0.01	0	0.01	0	0.01
Citral	13.07	1276	1276	0	0.01	0	0.02	0	0.02	0	0.02
(+)-Cyclosativene	14.67	1384	1368	0	0	0.01	0.01	0	0.01	0.01	0.01
Copaene	14.73	1388	1392	0.03	0.01	0.05	0.03	0.06	0.03	0.1	0.02
Eremophilene	16.34	1505	1503	0.01	0.01	0.01	0.01	0.01	0.01	0.01	0.01
alpha-Farnesene	16.39	1509	1509	0.05	0.03	0.06	0.03	0.05	0.03	0.05	0.07
Kessane	16.94	1551	1530	0	0	0	0	0	0	0	0
Liguloxide	17.07	1561	1533	0	0	0.01	0.01	0	0.01	0	0.01
**Others**											
2-Methylpentane	1.62	588	573	0.04	0.03	0.04	0.02	0.03	0.02	0.03	0.01
3-Methylpentane	3.39	741	748	0.05	0.03	0.07	0.03	0.08	0.03	0.09	0.03
Toluene	3.86	770	771	0.17	0.1	0.19	0.21	0.13	0.24	0.06	0.2
1-Octene	4.25	794	792	0.17	0.1	0.15	0.08	0.14	0.07	0.21	0.05
Octane	4.42	803	800	1.64	0.78	2.13	1.08	2.28	1	3.86	0.79
Styrene	6.36	898	899	0.04	0.04	0.04	0.06	0	0.06	0.09	0.11
o-Xylene	6.36	898	899	0.04	0.05	0.06	0.07	0.06	0.07	0	0.1
(Prop-2-en-1-yl)cyclopentane	6.31	895	898	0.31	0.06	0.27	0.07	0.23	0.08	0.22	0.06
Pentene dimer 1, C5	6.4	899	-	0.22	0.05	0.19	0.06	0.16	0.06	0.15	0.05
Pentene dimer 2, C5	7.19	940	947	1.65	0.41	1.41	0.45	1.21	0.47	0.98	0.33
Pentene dimer 3, C5	7.33	947	949	1.9	0.46	1.54	0.53	1.35	0.53	1.22	0.34
4,8-Dimethyl-1,7-nonadiene	7.38	949	998	0.01	0.02	0	0.01	0	0.01	0	0
Propylbenzene	7.59	960	962	0	0	0	0	0	0	0	0.02
2,2,6-Trimethyloctane	7.63	961	964	0	0	0.01	0.03	0	0.02	0	0.15
2,2-Dimethyl-3-heptanone	7.69	965	965	0.78	0.62	0.42	0.43	0.23	0.39	0.46	0.22
1-Ethyl-2-methylbenzene	7.73	971	969	0.05	0.03	0.04	0.04	0.03	0.04	0.04	0.05
5-Ethyl-2(5*H*)-furanone	7.82	971	968	12.91	12.72	5.39	8.7	2.22	7.76	6.5	3.27
2,2,4-Trimethylpentane	8.2	990	-	0.15	0.5	0.05	0.34	0	0.38	0	0.08
Pentene dimer 4, C5	8.27	994	-	0.88	0.23	0.77	0.23	0.66	0.24	0.58	0.18
Pentene dimer 5, C5	8.34	998	-	3.26	0.87	2.7	0.92	2.38	0.94	2.14	0.63
2-Propylfuran	8.45	1004	-	0.12	0.05	0.1	0.04	0.08	0.04	0.13	0.04
4-Ethyltoluene	8.89	1029	-	0.02	0.01	0.02	0.01	0.01	0.01	0.02	0.01
2,2,4,4,6-Pentamethylheptane	8.93	1030	-	0	0	0	0.01	0	0.04	0	0.07
5-Ethyl-2(5*H*)-furanone	9.17	1044	-	0.64	0.66	0.27	0.44	0.12	0.39	0.31	0.14
1-Phenyl-1-propanone	9.42	1059		0	0	0	0.01	0	0	0	0
1,4-Diethylbenzene	9.53	1063	1070	0	0	0	0.01	0	0.01	0	0
1-Undecene	10.07	1078	1082	0.04	0.01	0.03	0.02	0.03	0.02	0.02	0.02
(*E*)-4,8-Dimethyl-1,3,7-nonatriene	10.11	1096	1098	0.06	0.02	0.09	0.04	0.09	0.04	0.05	0.03
Methyl benzoate	10.27	1104	1106	0.04	0.01	0.05	0.02	0.05	0.02	0.03	0.01
Naphthalene	11.88	1201	1206	0.06	0.24	0	0.15	0.01	0.13	0	0
(*E*)-2-Dodecene	11.96	1206	1206	1.78	0.32	2.14	0.64	2.02	0.67	1.05	0.58
1-Dodecene	12.96	1270	1265	0.04	0.02	0.04	0.02	0.03	0.02	0.07	0.02
Pentadecane	16.28	1501	1500	0.01	0.01	0	0.01	0	0.01	0.02	0.01
2,4-Di-tert-butylphenol	16.44	1513	1517	0.02	0.01	0.02	0.01	0.01	0.01	0.01	0.01
Total				108.1		81.42		92.67		85.77	
C5				13.6		11.3		12.35		12.06	
C6				54.37		42.93		47.86		42.74	
C5 + C6				67.97		54.22		60.21		54.8	

Rt: Retention Time, I.S.: Internal Standard, RIex: Retention Index experimental, RIlit: Retention Index literature, SD: Standard Deviation.

**Table 3:** Factors and their corresponding levels in each portion of the RSM design: cube portion for the FF base design and star portion for the CCC augmentation.

Abbr.	Cube Level 1	Cube Level 2	Star Level 1	Star Level 2	Units
Ex.temp	40	70	25	85	°C
Co.time	10	20	5	25	mins
Ex.time	20	50	5	65	mins
Des.temp	250	270	240	280	°C
Des.time	5	10	2.5	12.5	mins

Ex.temp stands for extraction temperature, Co.time for conditioning time, Ex.time for extraction time, Des.temp for desorption temperature, and Des.time for desorption time. Units are standard SI units for each factor.

## Data Availability

Not applicable.

## Supplementary Materials

The following supporting information can be downloaded at https://www.mdpi.com/article/10.3390/metabo12020114/s1, Table S1: Quadratic model parameters including term estimates, standard error (Std.Error), t values and *p*-values. Term significance is provided in the column Signif, in a scale given at the footnote of the table. Extraction temperature (x1), conditioning time (x2), extraction time (x3), desorption time (x4) and desorption temperature (x5). Example: x1 is a first order term, x1:x2 is a two-way interaction term and x1^2 is a quadratic term, Table S2: Quality characteristics of EVOO samples, Table S3: Complete Circumscribed Central Composite design indicating the run order along with the levels of each factor. Units for extraction temperature (Ex.temp) are °C, conditioning time (Co.time) mins, extraction time (Ex.time) mins, desorption temperature (Des.temp) °C and desorption time (Des.time) mins, Figure S1: Contour plot for factors desorption temperature (Des.temp) and desorption time (Des.time). Green colour indicates lower response values and pink colour higher response values. Contours include corresponding response levels. The contour plot is a slice at the selected points for extraction temperature (Ex.temp) and extraction time (Ex.time), and the centre point for conditioning time (Co.time), Figure S2: PCA score plot showing the examined groups together with QC samples. QC samples are clustering together, showing system’s stability, Figure S3: Geographical origin of Cretan EVOOs analysed in our study.
