# Supplementary material for: Optimisation of the HS-SPME/GC-MS Approach by Design of Experiments Combined with Chemometrics for the Classification of Cretan Virgin Olive Oils
Source: Metabolites. 2022 Jan 25;12(2):114. doi: 10.3390/metabo12020114 (PMC8878322; doi:10.3390/metabo12020114)
Supplement: Supplementary file 1 [file metabolites-12-00114-s001.zip › metabolites-1565117-supplementary.pdf]

Supplemental Data

Optimisation of HS-SPME-GC-MS Approach by Design of Experiments Combined with Chemometrics for the Classification of Cretan Virgin Olive Oils

Artemis Lioupi[1,2,3], Ioannis Sampsonidis[2,3,4], Christina Virgiliou[1,2,3], Vasiliki Papoti[5], Apostolos Spyros[6], Kyriaki G. Zinoviadou[5], Georgios Theodoridis[1,2,3,*]

1 Laboratory of Analytical Chemistry, School of Chemistry, Aristotle University of Thessaloniki, 54124 Thessaloniki, Greece

2 Biomic AUTh, Center for Interdisciplinary Research and Innovation (CIRI-AUTH), Balkan Center B1.4, 10th km Thessaloniki-Thermi Rd, P.O. Box 8318, GR 57001, Thessaloniki, Greece

3 FoodOmicsGR Research Infrastructure, AUTh Node, Center for Interdisciplinary Research and Innovation (CIRI-AUTH), Balkan Center B1.4, 10th km Thessaloniki-Thermi Rd, P.O. Box 8318, GR 57001, Thessaloniki, Greece

4 Department of Nutritional Sciences and Dietetics, International Hellenic University, GR-574 00 Thessaloniki, Greece

5 Department of Food Science and Technology, Perrotis College, Thessaloniki, Greece

6 NMR Laboratory, Department of Chemistry, University of Crete, P.O. Box 2208, Voutes Campus, 710 03, Heraklion, Crete, Greece

* Correspondence: gtheodor@chem.auth.gr (G. Theodoridis)

Table S1: Quadratic model parameters including term estimates, standard error (Std.Error), t values and *p*-values. Term significance is provided in the column Signif, in a scale given at the footnote of the table. Extraction temperature (x1), conditioning time (x2), extraction time (x3), desorption time (x4) and desorption temperature (x5). Example: x1 is a first order term, x1:x2 is a two-way interaction term and x1^2 is a quadratic term.

	**Estimate**	**Std. Error**	**t value**	**Pr(>|t|)**	**Signif.**
**(Intercept)**	4848543089	48399344	100.1779	<2.2e-16	***
x1	177609148	24769154	7.1706	1.82E-05	***
x2	-13202358	24769154	-0.5330	0.6046238	
x3	127294342	24769154	5.1392	0.0003236	***
x4	40332181	24769154	1.6283	0.1317333	
x5	26088628	24769154	1.0533	0.3148037	
x1:x2	-40729724	30335894	-1.3426	0.2064436	
x1:x3	-3857485	30335894	-0.1272	0.9011092	
x1:x4	28416376	30335894	0.9367	0.3690108	
x1:x5	26551327	30335894	0.8752	0.4001486	
x2:x3	-20189553	30335894	-0.6655	0.5194209	
x2:x4	30561022	30335894	1.0074	0.3353792	
x2:x5	24270080	30335894	0.8000	0.4406207	
x3:x4	33310092	30335894	1.0980	0.2956397	
x3:x5	50072008	30335894	1.6506	0.1270555	
x4:x5	-58140167	30335894	-1.9165	0.0816296	.
x1^2	18949684	22404543	0.8458	0.4156859	
x2^2	-20693141	22404543	-0.9236	0.3755034	
x3^2	-39874246	22404543	-1.7797	0.1027255	
x4^2	-35067089	22404543	-1.5652	0.1458367	
x5^2	-15047133	22404543	-0.6716	0.5156883	

Signif. codes: '***' -0.001 '**'- 0.01 '*'-0.05 '.'-0.1

Table S2: Quality characteristics of EVOO samples.

No	Sample code	Variety	Type	Origin	Region
1	TN8	Koroneiki	Organic	Mesara	Heraklion
2	KX8	Koroneiki	Conventional	Rethymnon	Rethymnon
3	PK6	Koroneiki	Organic	Panormo	Rethymnon
4	ZA2	Tsounati	Conventional	Episkopi	Heraklion
5	ZK4	Koroneiki	Conventional	Kissamos	Chania
6	KP1	Koroneiki	Organic	Chania	Chania
7	AM9	Koroneiki	Organic	Manoliopoulo	Chania
8	XT2	Koroneiki	Conventional	Mesara	Heraklion
9	XA9	Koroneiki	Conventional	Kolimpari	Chania
10	XP5	Koroneiki	Organic	Peza	Heraklion
11	AT9	Koroneiki	Conventional	Maramvelos	Lasithi
12	ZB5	Koroneiki	Conventional	Agia Varvara	Heraklion
13	NT1	Koroneiki	Conventional	Choustouliana	Heraklion
14	BK5	Koroneiki	Conventional	Kolimpari	Chania
15	KN2	Koroneiki	Organic	Apokoronas	Chania
16	AN7	Koroneiki	Conventional	Mesara	Heraklion
17	XM4	Koroneiki	Conventional	Akrotiri	Chania
18	XB1	Koroneiki	Organic	Mesara	Heraklion
19	NX3	Koroneiki	Conventional	Mylopotamos	Rethymnon
20	AE5	Koroneiki	Conventional	Apokoronas	Chania
21	TM5	Koroneiki	Conventional	Kalesa	Heraklion
22	KM9	Koroneiki	Conventional	Kolimpari	Chania

23	NM6	Koroneiki	Organic	Mesara	Heraklion
24	NT9	Koroneiki	Organic	Chania	Chania
25	EO5	Koroneiki	Organic	Rethymnon	Rethymnon
26	AK5	Koroneiki	Conventional	Sitia	Lasithi
27	BN3	Koroneiki	Conventional	Arkalochori	Heraklion
28	AK1	Tsounati	Organic	Selino	Chania
29	PM2	Koroneiki	Conventional	Sitia	Lasithi
30	ZH3	Koroneiki	Organic	Apesokari Mesara	Heraklion
31	MN4	Koroneiki	Organic	Sitia	Lasithi
32	HN6	Koroneiki	Conventional	Ageliana	Rethymnon
33	MK2	Koroneiki/Tsounati	Conventional	Chania	Chania
34	NK8	Koroneiki	Conventional	Xerokampi	Heraklion
35	TA1	Koroneiki	Organic	Mesara	Heraklion
36	BO3	Koroneiki	Conventional	Mesara	Heraklion
37	OE3	Koroneiki	Conventional	Emparos	Heraklion
38	ΠΧ2	Koroneiki	Conventional	Heraklion/Chania	Heraklion/Chania
39	ZT6	Koroneiki	Conventional	Kolimpari	Chania
40	AT8	Koroneiki	Conventional	Mesara	Heraklion
41	HE2	Koroneiki	Organic	Laithi	Lasithi
42	TA5	Koroneiki	Conventional	Sitia	Lasithi
43	PB9	Koroneiki	Conventional	Zakros	Lasithi
44	TM8	Tsounati	Organic	Mariou	Rethymnon
45	HK3	Koroneiki	Conventional	Mesara	Heraklion
46	OZ7	Koroneiki	Organic	Kolimpari	Chania
47	KM4	Koroneiki	Conventional	Thrapsano	Heraklion
48	TΥ1	Koroneiki	Conventional	Rethymnon	Rethymnon
49	ZK1	Koroneiki	Conventional	Kritsa	Lasithi

50	NE1	Koroneiki	Conventional	Lyttos	Heraklion
51	TZ2	Koroneiki	Conventional	Sivas	Heraklion
52	NT5	Koroneiki	Conventional	Peza	Heraklion
53	HP2	Koroneiki	Conventional	Mylopotamos	Rethymnon
54	HT5	Koroneiki	Conventional	Kritsa	Lasithi
55	KT5	Koroneiki	Conventional	Kalesa malevisiou	Heraklion
56	MP1	Koroneiki	Organic	Anoskeli	Chania
57	ZE2	Koroneiki	Conventional	Mariou	Rethymnon
58	PT5	Koroneiki/Tsounati	Conventional	Heraklion/Chania	Heraklion/Chania
59	XM1	Koroneiki	Conventional	Emparos	Heraklion
60	TM4	Koroneiki	Conventional	Kritsa	Lasithi
61	MN8	Koroneiki	Organic	Ageliana	Rethymnon
62	AB3	Koroneiki	Conventional	Heraklion	Heraklion
63	EK2	Koroneiki	Conventional	Choumeri	Heraklion

Table S3: Complete Circumscribed Central Composite design indicating the run order along with the levels of each factor. Units for extraction temperature (Ex.temp) are °C, conditioning time (Co.time) mins, extraction time (Ex.time) mins, desorption temperature (Des.temp) °C and desorption time (Des.time) mins.

Run order	Ex.temp	Co.time	Ex.time	Des.temp	Des.time
1	55	15	35	260	7.5
2	55	15	35	260	7.5
3	40	20	20	250	5
4	70	20	50	250	5
5	40	10	20	270	5
6	70	20	50	270	10
7	40	20	50	250	10
8	40	20	50	270	5
9	40	10	20	250	10
10	70	10	20	270	10
11	55	15	35	260	7.5
12	55	15	35	260	7.5
13	70	20	20	270	5
14	70	10	50	250	10
15	40	10	50	270	10
16	70	10	20	250	5
17	70	10	50	270	5
18	40	20	20	270	10
19	70	20	20	250	10
20	40	10	50	250	5
21	55	15	35	260	7.5
22	55	15	35	260	7.5
23	55	15	35	260	12.5
24	25	15	35	260	7.5
25	55	25	35	260	7.5
26	55	15	35	240	7.5
27	55	15	35	260	2.5
28	55	15	35	280	7.5
29	85	15	35	260	7.5
30	55	15	5	260	7.5
31	55	5	35	260	7.5
32	55	15	65	260	7.5

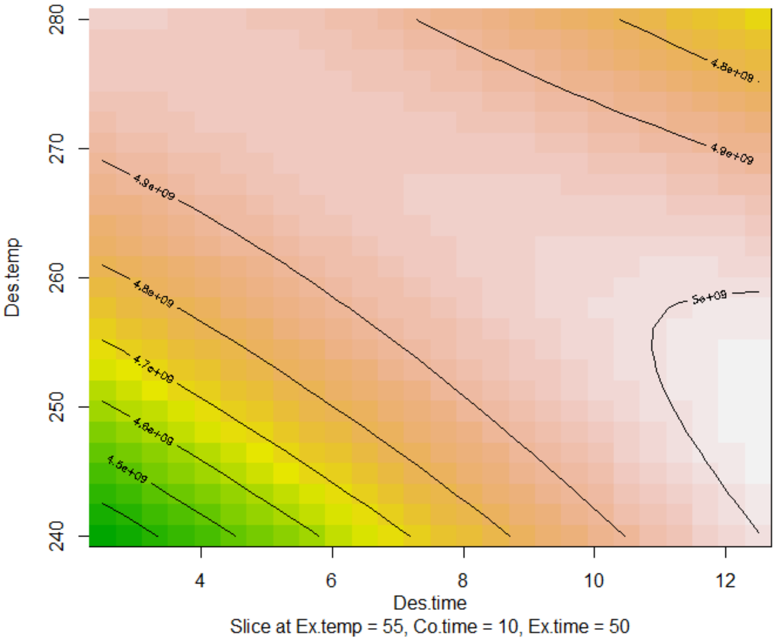

Figure S1: Contour plot for factors desorption temperature (Des.temp) and desorption time (Des.time). Green colour indicates lower response values and pink colour higher response values. Contours include corresponding response levels. The contour plot is a slice at the selected points for extraction temperature (Ex.temp) and extraction time (Ex.time), and the centre point for conditioning time (Co.time).

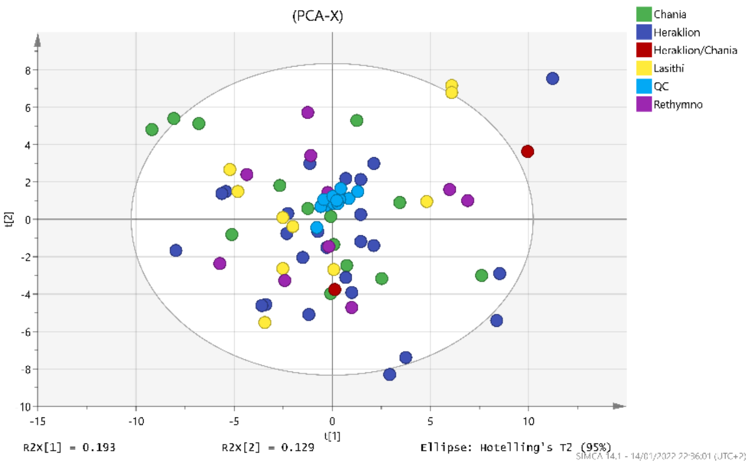

Figure S2: PCA score plot showing the examined groups together with QC samples. QC samples are clustering together, showing system's stability.

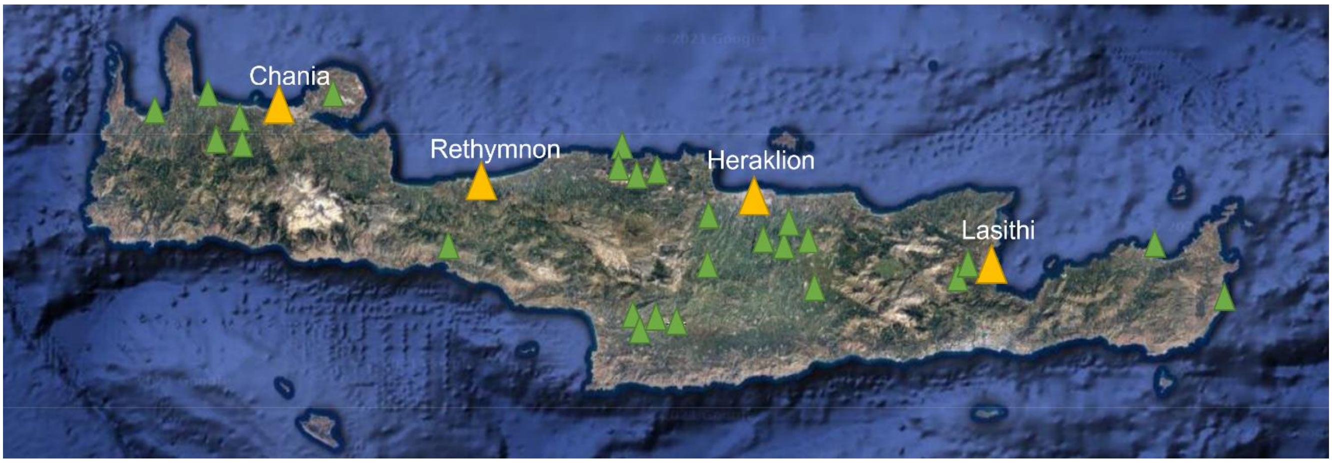

Figure S3: Geographical origin of Cretan EVOOs analysed in our study.
